# Experimental Analysis of Matrix Cracking in Glass Fiber Reinforced Composite Off-Axis Plies under Static and Fatigue Loading

**DOI:** 10.3390/polym14112160

**Published:** 2022-05-26

**Authors:** Gordon Just, Ilja Koch, Maik Gude

**Affiliations:** Institute of Lightweight Engineering and Polymer Technology, Technische Universität Dresden, Holbeinstr. 3, 01307 Dresden, Germany or ilk@mailbox.zih.tu-dresden.de (I.K.); maik.gude@tu-dresden.de (M.G.)

**Keywords:** fatigue, glass fiber, matrix cracking, polymer-matrix composites, transverse cracking

## Abstract

The inter-fiber failure of glass fiber-reinforced epoxy specimens with four different fiber angles was analyzed. Flat specimens were subjected to static and fatigue loading considering different load levels and load ratios. Damage investigation in terms of crack density measurement was performed by transmitted white light imaging using a digital camera and LED illumination from the back of the specimen on a servo-hydraulic testing machine. Static and fatigue results were examined with respect to crack initiation and crack growth, considering the special case of bonding yarns parallel to the fiber directions. The bonding yarns act as stress concentrations, influencing the early cracking behavior, and complicate the detectability of cracks growing underneath or next to the bonding yarns. In cyclic loading, the influence of load level, load ratio, mean stress, fiber orientation, and ply thickness was the focus of the experimental campaign. Cyclic cracking behavior in terms of initiation and growth was analyzed based on the applied loading conditions and laminate configurations. It was found that halving the ply thickness nearly doubled the amount of microcracks in case of high loads. For low loads, no such effect was observed up to $5\times 10^{5}$ loading cycles. Experimental findings on individual crack growth confirmed that crack interaction started for crack spacings less than four times the ply thickness and that subsequent crack growth shifted into regions of larger local crack spacing.

## 1. Introduction

The fatigue behavior of fiber-reinforced plastics (FRP) is of high interest for many technical applications due to the excellent material properties and low weight of the structural components. They are used in many industrial applications, both for structural load bearing purposes in naval or wind power sectors and for porous materials [1,2]. Many researchers have investigated the fatigue behavior of FRP based on the strength to number of cycles (SN) relationship, aiming to describe the final failure of the specimens due to uni- and bi-directional loading for several fiber orientations [3,4,5,6,7]. These experiments mainly describe the final failure of the composite, and do not capture the complex nature of progressive laminate failure. In order to gain a better understanding of the FRP failure process in composite laminates, the damage mechanisms themselves as well as their initiation and evolution have to be investigated.

It is known that an embedded ply forms multiple sub-critical cracks prior to final failure, and that these grow along the fiber direction through the entire width of the specimen [8,9,10,11,12,13]. Although this early-stage damage is not directly responsible for specimen failure, it is the reason for the major part of the stiffness loss that is observed during experiments. Furthermore, the cracks initiate subsequent failure mechanisms such as intra- and interlayer delaminations as well as fiber failure in adjacent layers. Hence, current research seeks to address the analysis and modelling of crack initiation and growth in embedded layers of bi- or multidirectional laminates [8,13,14,15,16,17,18,19,20,21,22,23].

Great effort has been put into the analysis of cross-ply laminates, which are a special case of fiber alignment rarely used for structural applications. However, the analysis of cross-ply laminates has yielded fundamental findings on the cracking process in FRP laminates, and a variety of models have been developed based on these findings [24,25,26,27], several of which have accounted for the statistical distribution of critical material properties [9,28,29,30].

Specifically, Berthelot [28] used two pseudo-normal strength distributions with limited domains of existence to describe the fracture strength in 90°-plies. The existence of initial flaws and defects was addressed by the use of a second distribution with very low strength values. In [29] Weibull, distributions with varying parameters were used to describe the cracking process in three different material systems with four different lay-ups, respectively. For each configuration, a unique parameter set for the fracture toughness distribution was found to describe the cracking process for static loading conditions. In [9,30,31,32], Weibull distributions were applied to SN-curves for crack initiation and to Paris-relations for crack growth in order to account for the stochastic nature of the fatigue cracking process within the models.

On the contrary, the amount of experimental data for the analysis of the cracking process in laminates comprised of layers with fiber angles $\theta_{f}<90{}^{\circ}$, in particular for fatigue loading, remains limited. In recent years, results for several fiber orientations have been published [14,15,16,23,30,31,32,33]. The main drawback is the complicated analysis of crack density in angle-ply laminates, which can be overcome by transmitted white light imaging (TWLI) and direction-dependent image filtering [16], which is only applicable to glass fiber reinforced plastics (GFRP), or by the use of tubular cross-ply specimens loaded by normal and shear stresses [34]. However, comparability of the results of different authors is questionable because of varying fiber–matrix combinations, different fiber volume fractions as well as different loading and testing conditions, e.g., bending vs. tension, different testing frequencies, etc.

For modeling purposes, it is essential to know what the driving forces of damage are and whether the model parameters have a physical meaning. Current models ([9,30,31,32,35]) use the maximum strain energy release rate $G_{max}$ to establish crack growth relations. However, they either do not implement or do not validate the influence of the load ratio [9,31,32,35], or the model accuracy greatly decreases [30]. Hence, it is of particular interest to identify how the influence of the load ratio can be appropriately considered in modeling. Furthermore, it has been observed that the model accuracy for high crack densities is not satisfactory for models relying on the assumption of equidistant crack spacing [36]. This drawback can be overcome by introducing a scaling parameter, *f*, that artificially increases the crack spacing used for calculations. It is therefore presumed that crack growth mainly takes place in areas with crack spacings greater than the equidistant distance, although this has not been experimentally verified.

The research presented here investigates the formation of microcracks within a non-crimped glass fiber-reinforced epoxy resin, dealing with the problem of bonding yarns parallel to the fiber direction, an issue which has not yet been addressed. These yarns influence the cracking process itself and complicate the process of crack detection, which can be overcome by advanced image processing. The presented work further aims to provide an explanation for the choice of the appropriate driving force for fatigue crack growth with respect to the load ratio using energy-based considerations, and supports the findings by experimental data. Additionally, the crack shielding effect encountered in laminate microcracking is quantified and the physical meaning of model scaling factors [36] is experimentally verified. Lastly, the investigation consistently analyzes both static and cyclic cracking behavior for a wide range of fiber angles, load levels, and load ratios, thus providing consistent data for the cracking process of both the material used here and for future modeling puposes.

## 2. Experimental Procedure for Matrix Crack Density Measurements

### 2.1. Materials and Specimen Preparation

The glass fiber-reinforced polymer laminates investigated in this research were made of Sigratex GU220-0/SO unidirectional (UD) E-glass consisting of dry fabrics with an areal weight of 220 g/m^2^ bonded on a single-side by polyester yarns. As matrix material, an epoxy resin system RIMR135 in combination with a hardener RIMH137 at a mixing ratio of 100:30 was used. The laminate lay-ups were stacked manually and consisted of twelve individual plies. The dry stacks were infiltrated in terms of resin transfer molding (RTM) and cured at 80 °C for 15 h. To alleviate variations in the glass transition temperature, $T_{g}$, the laminates were tempered for an additional 4 h at 80 °C after demolding.

The UD-laminates were used for the determination of the material’s elastic and strength properties on a Zwick 1465 universal testing machine equipped with a 50 kN load cell and traveling extensometer for strain measurement. Specimens were cut from the cured composite plates by water jet cutting. While the edges were not polished after cutting, no undesired initial cracks or delaminations due to the cutting process were observed during visual inspection. The specimens were tested for axial tension and for in-plane shear in terms of the V-notched rail shear (VNRS) according to the applicable standards. Aluminium end tabs with a 45°-chamfer towards the gauge length were bonded to the UD specimens using Scotch Weld DP-490 epoxy-based adhesive to minimize clamping effects, except for the VNRS specimens, where tabs are not mandatory. The results of the material characterization in terms of the mean value, μ, standard deviation, σ, and number of experiments, $n_{exp}$, are provided in Table 1.

Furthermore, laminates with four different lay-ups (see Table 2) were manufactured in order to determine the laminate properties and crack growth behavior during internal multiaxial stress states under static and fatigue loading. The multiaxial stress states within the embedded plies arise, even in case of uniaxial external loading, due to fiber orientation and anisotropic material behavior. These fiber orientations were chosen in order to cover a wide range of fiber angles, and therefore different stress states, as well as to reasonably complement the existing data.

**Figure 1:**
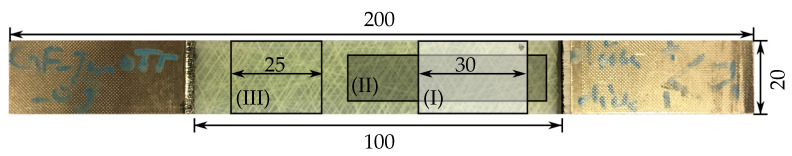
Dimensions (in mm) of the specimens used for static and fatigue analysis and the areas used for (I) matrix crack counting, (II) thermographic imaging, and (III) strain measurement within the specimen gauge length at the front side.

In accordance with the UD tensile tests, the laminates were equipped with aluminium end tabs as well. The specimens were cut to their final dimensions using a high-precision abrasive cutting machine, and the edges were not polished after cutting. An image of a representative specimen used for matrix cracking analysis with its final dimensions is shown in Figure 1 for a $\left[0/{+55}_{2}/0/{-55}_{2}\right]$ laminate specimen. The bonding yarns are clearly visible and the areas used for (I) cracking analysis, (II) thermographic imaging, and (III) strain measurement at the front side of the specimen are indicated.

In contrast to the UD material characterization, static and fatigue laminate tests were performed on a servo-hydraulic testing machine (see Section 2.2). Details on the stacking sequence, laminate thickness $t_{lam}$, and fiber volume fraction $\varphi_{f}$, as well as the results of the tensile tests in terms of axial stiffness $E_{xx}$ and strength $R_{xx}$, are summarized in Table 2.

The number of 0°-plies was equal for all laminates. However, in the case of multiaxial lay-ups the 0°-plies were placed between the +θ and −θ layers to prevent interaction of cracks within the layers and triggering of microcrack initiation due to stress concentrations in the layer interface.

### 2.2. Experimental Setup for Microcracking Analysis

The crack counting experiments were conducted on an Instron 8801 servo-hydraulic testing machine equipped with a 100 kN load cell and mechanical wedge grips. The static experiments were carried out in terms of displacement control, whereas the fatigue experiments were performed by means of load control. An extensometer with a gauge length of $l_{0}=25\;mm$ was used to determine the applied strains. Additionally, a thermographic camera was used to monitor possible specimen heating throughout the experiments for selected specimens exhibiting high stress amplitudes. The measurements revealed a maximum temperature increase of $\Delta T_{max}\approx 5K$ when the cooling fan was in use. For the case of compressive stresses within a load cycle, a linear variable differential transformer (LVDT) was mounted perpendicular to the specimen surface to monitor possible specimen bending.

**Figure 2:**
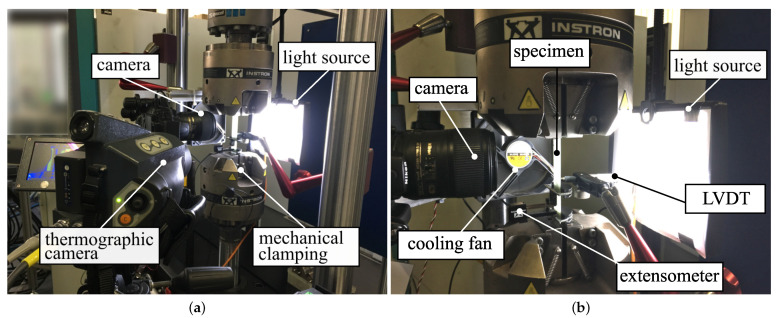
Experimental setup used for crack analysis in static and fatigue loading: (**a**) complete setup and (**b**) detail of the specimen and measuring equipment.

Damage characterization was performed in terms of automated crack counting. The experimental setup used for the evaluation of matrix cracking is shown in Figure 2.

Based on the presented detection algorithm in Section 3, many static and cyclic experiments were performed in order to analyze the crack density evolution in angle-ply laminates. Initially, our interest was in determining when the first cracks occurred within the laminates. Therefore, crack initiation is defined here by the crack density $c_{ini}=0.075{\;mm}^{-1}$, which corresponds approximately to the length of two cracks spanning the total specimen width. This threshold was chosen to overcome minor uncertainties in the crack detection process arising from environmental influences or from the procedure itself.

In order to determine the static initiation stress for matrix cracking, $\sigma^{i}$, the specimens were statically loaded up to an intended load level and then unloaded to a stress level of $\sigma_{xx}^{lam}=50\;\mathrm{MPa}$ to obtain the image corresponding to the applied load level. This loading–unloading–reloading (LUR) procedure was repeated for several load levels until final specimen failure. An initial image without any damage was taken at the beginning of each test at the same stress level of $\sigma_{xx}^{lam}=50\;\mathrm{MPa}$.

The static experiments primarily served to analyze the cracking behavior under static loading conditions and to define the fatigue stress levels for the cyclic experiments. Each lay-up was tested at four different load levels for any of the three stress ratios. The maximum applied stress, $\sigma_{max}^{fat}$, for each load level was defined to be well below the static stress for microcrack initiation in order to avoid crack initiation due to static overstressing within the first few cycles, and corresponded to 90%, 77%, 63%, and 50% of $\sigma^{i}$ for all fiber orientations, respectively. Due to the expected very low crack growth in the cases of $\sigma_{max}^{fat}=0.5\times \sigma^{i}$ and R=0.5, the load level was replaced by $\sigma_{max}^{fat}=0.85\times \sigma^{i}$, as indicated in brackets in Table 3. A summary of the testing schedule, the stresses $\sigma_{22}$ and $\tau_{12}$ (calculated by means of classical lamination theory), the stress multi-axiality $\lambda_{12}=\left|\tau_{12}/\sigma_{22}\right|$, as well as the residual stresses $\sigma_{22,res}$ and $\tau_{12,res}$ within the off-axis plies (which were determined according to the procedure presented in [17]), are provided in Table 3. Due to the presence of residual stresses, the value of $\lambda_{12}$ depends on the applied load level and is not constant for a particular fiber orientation.

Considering the fatigue experiments, the tests were automatically paused ten times within every decade of load cycles in order to take the images. The specimens were not unmounted from the testing machine and the experiments were continued automatically after the image was taken. In accordance with static testing, an initial image was taken before any cyclic loading was applied. Consequently, a set of 41 images per fatigue test was analyzed by the proposed detection method. To determine the crack density, $c_{k}^{\theta }$, within the θ layer in every *k*-th image, the sum of the crack lengths was normalized by the area of observation in terms of

(1) $$c_{k}^{\theta }=\frac{{\left(\sum_{i=1}^{m}l_{i}^{\theta }\right)}_{k}}{A_{O}},$$

with the length of an individual crack $l_{i}^{\theta }$ in mm and the rectangular area of observation, $A_{O}$, in mm^2^. The definition of crack density used here is therefore in accordance with previous research [8,9,16].

The results of the experiments obtained by automated crack detection provide insight into the general influence of fiber orientation, layer thickness, and load level on the cracking process in terms of cyclic loading. Further details of the analysis and evaluation of the matrix cracking state by TWLI are provided below.

## 3. Matrix Crack Detection by Transmitted White Light Imaging

### 3.1. Crack Detection Procedure

One goal of the presented research is to facilitate and partially automate the data acquisition process for counting matrix cracks during static and fatigue experiments. Compared to manual analysis, this is advantageous because of the user-independent quantification of the crack density, potential reductions in the time required for experiments, more precise measurement of data, and prevention of possible misalignment due to repeated specimen removal and mounting.

**Figure 3:**
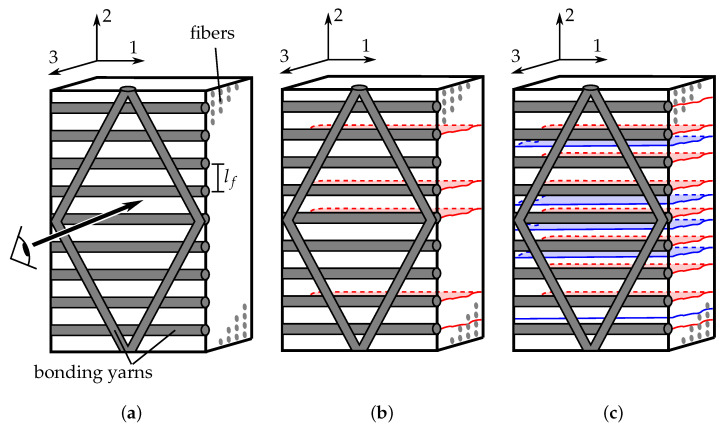
Schematic representation of the cracking process in a single layer of a given laminate: (**a**) the bonding yarns are adhesively attached on top of the UD-ply ($l_{f}=5\;mm$), (**b**) first crack initiation (red) covered by bonding yarns, and (**c**) subsequent initiation of additional cracks (blue) in the latter course of the experiment growing between bonding yarns, which are clearly visible. The direction of observation is parallel to the 3-direction.

The image acquisition procedure is based on transmitted white light imaging (TWLI), which is only applicable to transparent materials, e.g., glass fibers with a translucent resin. Several approaches for automated matrix crack counting in translucent materials have already been reported in the literature [8,16,19,37,38]. However, none of them dealt with cracks growing along the bonding yarns of the reinforcing textile, which may be aligned parallel to the fibers. In the presented research, the detection of matrix cracks growing along the bonding yarns is of particular interest because of early crack initiation (low loading or low number of cycles) compared to cracks in undisturbed areas. These early cracks can hardly be seen without advanced image processing techniques, as schematically depicted in Figure 3. The applied image acquisition and processing technique incorporates to successfully extract the matrix cracks from the images for each layer individually. Details of the developed crack counting methodology are provided below.

1. Image acquisition
2. Displacement compensation and image normalization
3. Anisotropic image filtering
4. Thresholding and crack measurement

#### 3.1.1. Image Acquisition

**Figure 4:**
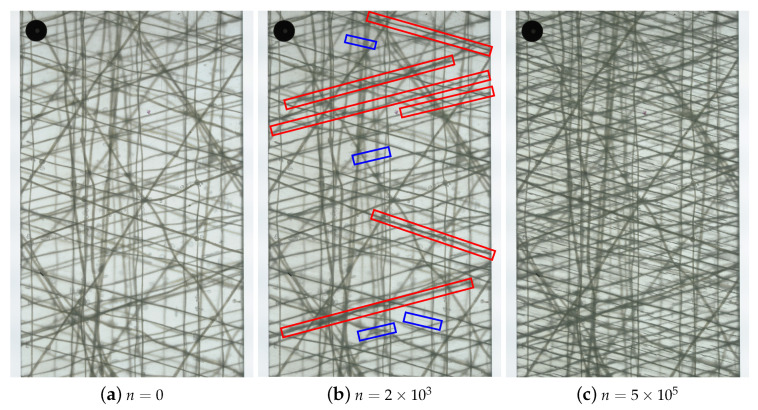
TWLI images of a ${\left[0/{+75}_{2}/0/{-75}_{2}\right]}_{s}$-laminate (**a**) at the very beginning of the test, (**b**) after $n=2\times 10^{3}$ load cycles, and (**c**) at the end of the experiment after $n=5\times 10^{5}$ cycles. In (**b**), cracks behind the bonding yarns are highlighted in red and subsequent cracks that are developing are highlighted in blue.

In the course of the cracking process, internal surfaces are generated which deflect transmitting light. The cracks appear as dark lines, whereas undamaged regions remain bright. Unfortunately, bonding yarns, which can act as crack nuclei, and matrix cracks may grow along or underneath them, appear darker than the surrounding crack free areas as well. In this particular case, it is nearly impossible to detect the matrix cracks from a single image by either observation with the naked eye or by conventional image processing methods, as depicted in Figure 4. It is therefore essential to highlight the matrix cracks and attenuate the bonding yarns through the use of a reference image taken at the very beginning of each test.

To improve the general visibility of the cracks, the images must be taken while the specimens remain loaded in tension during static and fatigue loading, respectively, to ensure opening of the cracks. As a consequence, the cracks appear wider, and even small cracks are visible. The corresponding reference images were taken at the same level of tension loading.

#### 3.1.2. Displacement Compensation and Image Normalization

The specimens elongate in the course of the experiments due to specimen damage and creep as well as to uncertainties in the control loop of the testing machine, leading to residual displacements. Using image compensation, the corresponding displacements of related pixels within the image, $I_{1}$, can be eliminated in order to compare local areas with the corresponding areas in the reference image, $I_{0}$.

Pixel-based displacement compensation is performed for the entire image instead of for distinct areas, and does not require the identification of distinct features within the field of view as long as displacements are small. Therefore, a non-parametric image registration technique, proposed by Thirion [39] and already implemented in Matlab (available as of Matlab R2014b) is used to estimate the required transformation. While the details of the image matching process are out of the scope of this study, they can be found in [39,40]. The displacement compensation is performed for each pixel iteratively. As a result, the matched image, $I_{1}^{\prime }$, as well as the displacement field, $\bar{D}$, in the *x*- and *y*-direction for each pixel within an image $I_{1}$ can be obtained. Hence, by additively applying the displacements $D_{x}\left(x,y\right)$ and $D_{y}\left(x,y\right)$ to a pixel in $I_{1}$, the new location of the pixel in the transformed image $I_{1}^{\prime }$ can be determined. In absence of damage, the intensity of the matched image, $I_{1}^{\prime }$, is now equal to the intensity of the reference image, $I_{0}$, at the same (x,y)-coordinates. Thus, a change in the intensity indicates matrix crack formation.

Finally, the matched image, $I_{1}^{\prime }\left(x,y\right)$, is normalized by the reference image, $I_{0}\left(x,y\right)$, yielding the normalized image, $I_{n}\left(x,y\right)$, according to

(2) $$I_{n}\left(x,y\right)=\frac{I_{1}^{\prime }\left(x,y\right)}{I_{0}\left(x,y\right)}.$$

**Figure 5:**
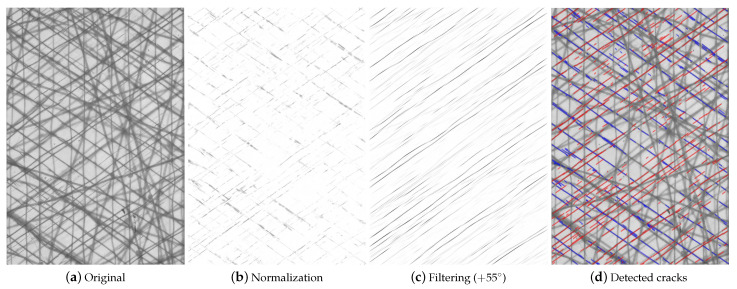
(**a**) Original image of a cracked specimen after $n=5\times 10^{4}$ loading cycles and results of the proposed image processing technique after (**b**) normalization and (**c**) Gabor filtering perpendicular to the +55°-direction. Detected cracks within the −55° (blue) and +55° layers (red) are plotted together with the original image in (**d**).

As a result, differences between the images, e.g., matrix cracks and delaminations, are highlighted, whereas similar regions, e.g., bonding yarns and crack-free areas, are attenuated, as depicted in Figure 5b.

#### 3.1.3. Filtering and Crack Detection

In order to distinguish between cracks in different orientations, the images are filtered with respect to the fiber direction. Gabor filtering [16,37,41] has been successfully used and is implemented in the presented crack detection procedure. The required parameters are the filter direction $\theta_{G}=\theta -90{}^{\circ}$, wavelength λ, spatial frequency bandwidth $\rho_{G}$, and the spatial aspect ratio β, which specifies the ratio of the semi-minor and semi-major axis of the filter envelope (the ellipticity of the filter). These parameters are calibrated for a representative set of images of the analyzed laminates, and can be further used for all specimens of the same material with comparable lighting conditions. The calibrated filter parameters and image specifications of width $W_{I}$, height $H_{I}$, and spatial resolution γ for the investigated laminates are summarized in Table 4.

The Gabor filter emphasizes discontinuities within the filtered images; hence, it highlights the cracks that are perpendicular to the filtering direction. Regarding specimens with two layers in the same orientation (+θ-direction in the present case), the cracks in the second +θ layer, which is closer to the back of the specimen, are not captured by the algorithm due to their very low contrast. Hence, the cracks counted by this procedure always refer to the layer which is closer to the camera and represent the crack density in a single thin off-axis layer.

In order to separate the cracks from the image background, a thresholding procedure based on the grayscale values within the magnitude images is applied to obtain a black and white image. After normalizing the pixel values of the magnitude images to a range of 0 to 1, the cracks are determined by setting the threshold to 10% of the maximum value. All pixels with lower values are treated as matrix cracks. Other thresholding methods, e.g., Otsu’s method [42], might be applicable as well. However, Otsu’s method defines an individual threshold for each image and might change with increasing amount of matrix cracks. From the authors’ point of view, a change in the threshold value depending on the crack density is not reasonable, and such a detection procedure might omit small cracks in the latter course of the experiments.

Finally, the individual cracks have to be extracted from the black and white images. Glud et al. [16] used a pixel-based method to evaluate the crack length, which is not applied here because the cracks cannot be reliably reduced to lines with a thickness of one pixel. Although the cracks are not perfectly straight, inevitably, the deviations are small and the extent of a crack $l_{i}$ can be approximated by evaluating the euclidean distance between the crack tips for each white area within the image. Therefore, the major and minor axis lengths of all remaining white areas are extracted by making use of the *regionprops()* function in Matlab, and the individual crack length is calculated from the major axis length divided by γ. An example of the proposed crack detection procedure for a ${\left[0/{+55}_{2}/0/{-55}_{2}\right]}_{s}$ laminate is shown in Figure 5.

The aforementioned procedure is performed for every image taken throughout the experiment with a fixed set of parameters that is calibrated once. All images are evaluated in the same way, ensuring comparability between different experiments. The validity and sensitivity of the developed procedure are checked in the next section.

### 3.2. Experimental Validation

It is not possible to validate the procedure by manual crack counting from the original images because of crack propagation along the bonding yarns, which is nearly invisible to visual inspection. In this case, manual crack counting would lead to erroneous results and crack densities might be greatly underestimated. Hence, manual crack counting was carried out by means of the normalized images, where the cracks are visible more clearly. Additionally, micrographic imaging was used to prove the growth of cracks along the bonding yarns.

**Figure 6:**
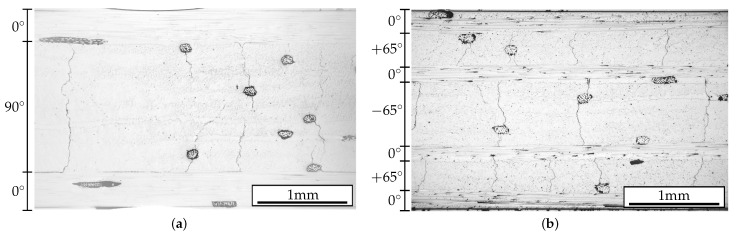
Edge micrographs of (**a**) ${\left[0_{2}/90_{4}\right]}_{s}$ and (**b**) ${\left[0/{+65}_{2}/0/{-65}_{2}\right]}_{s}$ laminate after fatigue testing for $n=5\times 10^{5}$ loading cycles. The dark cylindrical and elliptical areas represent bonding yarns. In (**a**), delaminations occurred during testing at the layer interfaces, emanating from the tips of the transverse matrix cracks.

First, the cracks were analyzed along the specimen edges for certain specimens. The specimen edges were polished after testing and micrographs were taken from the whole specimen edges along the area of observation. To avoid crack closure due to the polishing process, the specimens were cut in two parts and embedded in coloured resin for polishing. As illustrated in Figure 6, the cracks are clearly visible at the polished edges.

The number of cracks counted along the specimen edge cannot be directly compared to the crack density evaluated by TWLI, because for angle-ply laminates the crack direction is not perpendicular to the specimen edge, and it is not clear whether the cracks span the full specimen width. However, the micrographs in Figure 6 reveal that the cracks likely initiate next to the bonding yarns, which was expected due to the influence of weak interfaces or local stress concentration effects induced by the bonding yarns. Furthermore, the micrographs confirm that a change of the intensity of the bonding yarns within the images represents the initiation or growth of a matrix crack, and is therefore not a result of changing illumination conditions.

**Figure 7:**
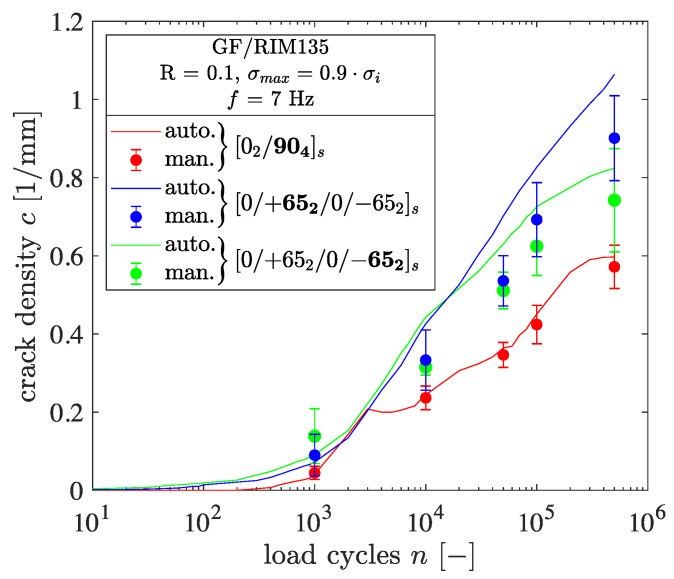
Comparison of manually and automatically detected cracks in ${\left[0_{2}/90_{4}\right]}_{s}$- and ${\left[0/{+65}_{2}/0/{-65}_{2}\right]}_{s}$-laminates, respectively.

In a second step, the cracks within two different laminates were counted manually from the normalized images to further check the accuracy of the algorithm. Therefore, five normalized intensity images, $I_{n}$, for two specimens with different fiber orientations were analyzed manually. The moments of observation were chosen to be spread along the whole number of cycles in order to check the algorithm’s accuracy for a wide range of crack densities. The comparison between the results obtained by manual and automated crack counting is provided in Figure 7. In the case of the ${\left[0/{+65}_{2}/0/{-65}_{2}\right]}_{s}$ laminate, the results are provided in terms of the thin +65° and thick −65° layers, respectively.

It should be noted that the task of manual crack counting is cumbersome and significantly affected by the user’s expertise. Very good agreement between manual and automated crack counting was achieved in the case of a cracked 90° layer for all of the five analyzed images. Regarding the thin and thick layers within the ${\left[0/{+65}_{2}/0/{-65}_{2}\right]}_{s}$ laminate, the agreement is good as well. The better agreement in the case of 90° off-axis plies might be due to more pronounced crack openings while the picture is taken under the applied load, meaning that cracks are easier to find for both user and algorithm.

In the case of ${\left[0_{2}/90_{4}\right]}_{s}$ and $\left[0/{+55}_{2}/0/{-55}_{2}\right]$, delaminations occur throughout the experiment that are currently not recognized by the detection algorithm. Delamination areas are not mistakenly detected as cracks due to their planar shape and low contrast. Consequently, the delaminations do not affect the crack counting results. Due to the noticeably good agreement between manual and automated crack counting, the accuracy of the algorithm is judged to be suitable for detailed analysis of the cracking behavior.

## 4. Results and Discussion

### 4.1. Static Loading

**Figure 8:**
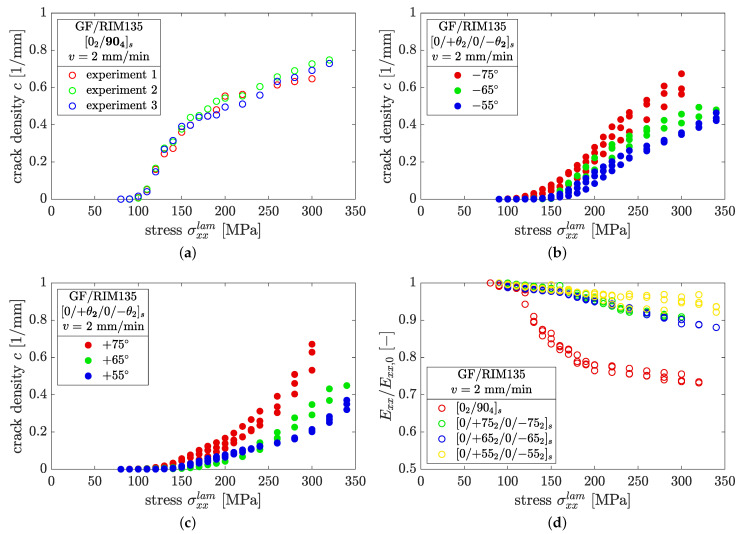
Results of crack density formation in (**a**) ${\left[0_{2}/90_{4}\right]}_{s}$ laminates, (**b**) the thick layers, and (**c**) the thin layers of angle-ply laminates; (**d**) shows the corresponding stiffness reduction for all laminates under static loading.

The results of the static LUR procedure are shown in Figure 8. All tested specimens show a significant amount of cracks before final specimen fracture. From Figure 8a, significant crack initiation in the case of ${\left[0_{2}/90_{4}\right]}_{s}$ laminates is observed from $\sigma_{xx}^{lam}\geq 110\;\mathrm{MPa}$, with a steep increase in crack density to $c\approx 0.4{\mathrm{mm}}^{-1}$ at $\sigma_{xx}^{lam}=150\;\mathrm{MPa}$. With further load increase the slope of the crack density evolution flattens, and the maximum crack density is $c\approx 0.73{\mathrm{mm}}^{-1}$ prior to final failure.

None of the specimens showed a crack density higher than $c\approx 0.8{\mathrm{mm}}^{-1}$. The lowest maximum crack densities were observed in the case of ${\left[0/+55_{2}/0/-55_{2}\right]}_{s}$ laminates, which remain below $c<0.5{\mathrm{mm}}^{-1}$ for all specimens, and for thick as well as thin layers. In cases of both thick and thin layers the crack density evolution increases for higher fiber angles, which can be attributed to higher normal loads and therefore to a higher mode I contribution to the cracking process.

Figure 8a–c shows the results for three different layer thicknesses, corresponding to $t_{90}=8t$, $t_{-\theta }=4t$, and $t_{+\theta }=2t$ with t≈0.18mm, respectively. Considering the slopes of the crack density curves, a change from declining (Figure 8a) to nearly linear (Figure 8b) to exponential (Figure 8c) can be observed. This phenomenon is attributed to the diminishing crack shielding effect as a direct consequence of the decreasing layer thickness [28].

The same behavior can be observed when analyzing the stiffness degradation due to crack formation, as depicted in Figure 8d. In the case of embedded 90°-plies, the greatest stiffness reduction, approximately 25%, was measured. The main part of the stiffness reduction takes place from $\sigma_{xx}^{lam}=110\;\mathrm{MPa}$ to 160 MPa and decreases subsequently. For laminates containing ±75°, ±65°, or ±55° plies, the maximum stiffness reduction was determined to be approximately 10%, 12%, and 7.5%, respectively, prior to final specimen failure, and appears to be somewhat insensitive to the fiber orientation, as previously observed by Fikry et al. [15].

From the static LUR experiments, the stress for crack initiation, $\sigma^{i}$, was determined for every fiber angle. In the following, the fatigue load levels were chosen to be well below the initiation stress, as summarized in Table 3.

### 4.2. Fatigue Loading

#### 4.2.1. Fatigue Crack Initiation

**Figure 9:**
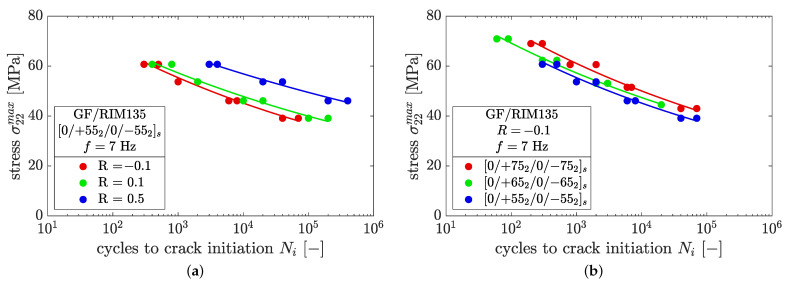
SN-curve for crack initiation in GF/RIM135 laminates in terms of maximum normal ply stress, highlighting the effects of (**a**) different load ratios and (**b**) different fiber orientations.

Following Carraro et al. [14] the fatigue behavior can be characterized by crack initiation and crack evolution. The cycles of crack initiation are shown in Figure 9 in terms of the maximum normal ply stress $\sigma_{22}^{max}$, where the circles represent experimental data and the lines refer to a power law relation of type $\sigma_{22}^{max}\;=\;CN_{i}^{m}$. Regarding the case of the ${\left[0/+55_{2}/0/-55_{2}\right]}_{s}$ laminate in Figure 9a, the results show that lower stress ratios lead to earlier crack initiation, which can be attributed to higher stress amplitudes. The trend of earlier crack initiation for decreasing stress ratios was consistently found for all fiber orientations.

The detrimental effect of shear stresses on crack initiation, as discussed in [14,34], was observed here as well. The presence of shear stresses leads to earlier crack initiation with a decreasing fiber angle, as seen in Figure 9b. This effect was consistently observed for all load ratios and might further increase in cases of multiaxial external loading. However, no significant influence of the ply thickness on crack initiation was observed, which is in accordance with findings in [9].

#### 4.2.2. Fatigue Crack Evolution

**Figure 10:**
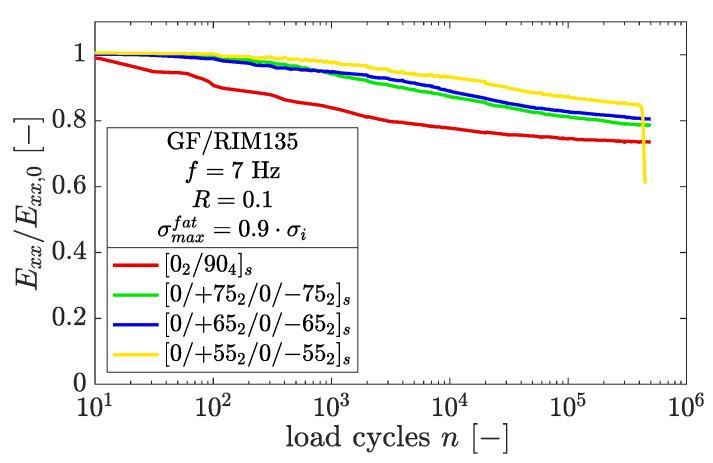
Stiffness loss for the tested laminates at R=0.1 and the highest load level, $\sigma_{max}^{fat}=0.9\times \sigma_{i}$.

In Figure 10, the differences in the stiffness loss for the laminates tested with R=0.1 and $\sigma_{max}^{fat}=0.9\times \sigma_{i}$ are plotted. The stiffness loss is slightly higher for increasing fiber angles, although the corresponding crack densities within the off-axis layers are not necessarily higher.

The highest stiffness loss was measured in the case of 90° off-axis layers, whereas the lowest stiffness loss was found for the ${\left[0/+55_{2}/0/-55_{2}\right]}_{s}$ specimen. The latter failed after *n* = 452,062 load cycles due to widespread delamination and subsequent ply splitting of the 0° layer. All other specimens represented in Figure 10 showed delamination growth, which was most pronounced in the case of the ${\left[0_{2}/90_{4}\right]}_{s}$ specimen. The delaminations mainly began growing from the specimen edges, and always in the presence of a microcrack.

**Figure 11:**
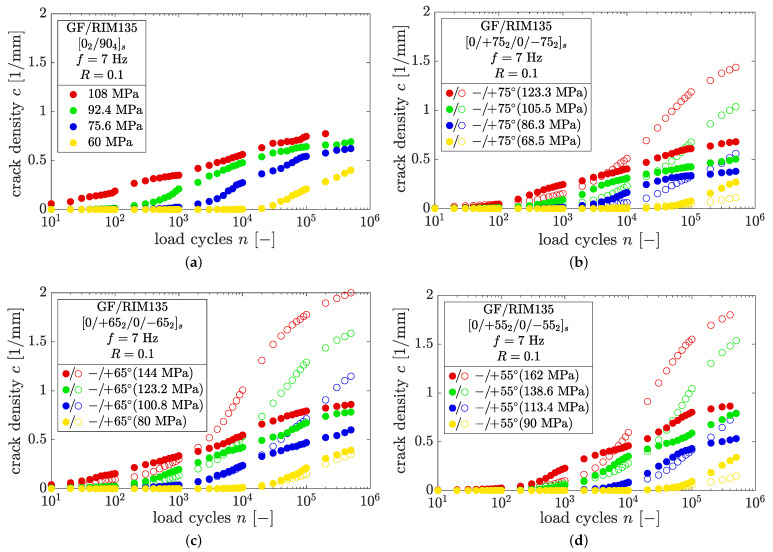
Crack density growth vs. number of cycles at R=0.1 for laminate lay-ups (**a**) ${\left[0_{2}/90_{4}\right]}_{s}$, (**b**) ${\left[0/+75_{2}/0/{-75}_{2}\right]}_{s}$, (**c**) ${\left[0/+65_{2}/0/{-65}_{2}\right]}_{s}$ and (**d**) ${\left[0/+55_{2}/0/{-55}_{2}\right]}_{s}$ under fatigue loading.

In Figure 11a–d, the results of the crack counting procedure in thin and thick plies as well as the dependence on the fiber orientation are shown for the load ratio R=0.1. The results of all the experiments at R=0.5 and R=−0.1 can be found in Appendix A.

**Figure 12:**
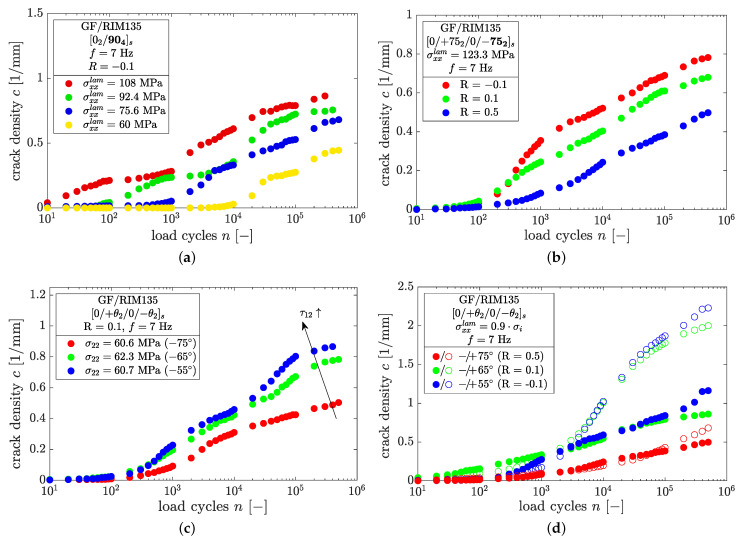
Influence of (**a**) load level, (**b**) load ratio, (**c**) fiber orientation, and (**d**) ply thickness on the formation and growth of microcracks under fatigue loading.

The crack density evolution is very sensitive to a high number of influencing factors. The effects of the load level, load ratio, fiber orientation, and ply thickness are summarized in Figure 12.

From Figure 12a, it can be seen that higher load levels lead to pronounced crack growth in the fatigue experiments, which is an effect of higher stresses and the resulting higher strain energies within the off-axis plies. This holds for all load levels, stress ratios, and fiber orientations, and is well documented in the literature [8,9,14,16,19,28,30].

**Figure 13:**
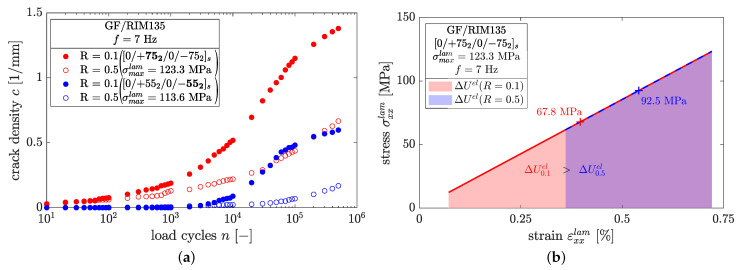
(**a**) Influence of mean stress on cyclic crack growth and (**b**) comparison of the range of the strain energy at different load ratios.

Regarding the influence of the load ratio, it was found that high load ratios cause delayed crack growth and lead to lower crack densities at the end of the tests (cf. Figure 12b). Throughout all experiments, negative load ratios led to the highest crack densities, which might be a consequence of the higher amplitudes and load reversals. The relationship c∝1/R was found for all fiber orientations and load levels. This can be explained by looking more closely at the strain energy within a load cycle. In Figure 13a examples are provided for laminates containing ±55° and ±75° plies which were loaded by the same maximum laminate stress but different load ratios, and thus have different amplitudes and mean stresses.

In both cases, specimens having a load ratio of R=0.5 show less cracks than the corresponding specimens at R=0.1. It is therefore reasonable to assume that crack growth under fatigue loading is driven by amplitude rather than maximum loads. This can be clarified by considering the range of the elastic strain energy, $\Delta U^{el}$, stored within the laminate throughout cyclic loading. The stress–strain relationship of a laminate comprised of ±75° layers at the beginning of a fatigue test is depicted in Figure 13b. For simplicity and because of the low stress levels applied in fatigue loading, the stress–strain relationship is assumed to be linear. The strain energy is provided by the area under the stress–strain curve and the abscissa. Both specimens shown in Figure 13a were loaded with the same maximum stress and different load ratios. It is evident that the range of the strain energy, $\Delta U^{el}$, which is related to the strain energy release rate according to G=−∂U/∂A, is smaller in case of R=0.5 compared to R=0.1. Hence, pronounced crack growth should expected for R=0.1. From the authors’ point of view, it is therefore reasonable to establish crack propagation laws in terms of the range of the total strain energy release rate, Δc/Δn vs. $\Delta G_{I+II}$. On the other hand, $G_{max}$ should not be considered as the driving force for crack growth due to its insensibility for, e.g., different load ratios in case of same maximum applied load. In such a case, the different crack density results in Figure 12b could not be explained.

The detrimental effect of additional shear stresses on the initiation and growth of cracks can be seen from Figure 12c, and has been found by previous authors as well [14,16,34]. In axial loading, shear stresses arise with decreasing fiber angle and cause pronounced crack growth for otherwise similar normal ply stresses of $\sigma_{22}$; ±55° plies exhibit the highest shear stresses. Interestingly, this is in contrast to the cracking behavior under static loading conditions, where low fiber angles delay crack initiation and generate a lower number of cracks prior to specimen failure (cf. Figure 8b,c).

Variations of the ply thickness affects the growth of microcracks within cyclic tests as well. From Figure 11b–d and Figure 12d it is clear that thin plies tend to generate more cracks, which was observed for all fiber orientations. A significant difference between thick and thin plies was found in the later course of the experiment, whereas for a crack density of $c<0.4{\mathrm{mm}}^{-1}$ the crack density evolution appears to be very similar in thin plies compared to thick plies. Regarding the architecture of the fiber material, this may be an influence of the bonding yarns, which have a distance of approximately 5 mm to each other. However, the difference between thin and thick plies diminishes with increasing load ratio as well as with decreasing maximum load, and might therefore be affected by the applied loading amplitude.

#### 4.2.3. Crack Interaction

It is known from the literature that adjacent cracks begin to interact when their distance is small [14,24]. Using TWLI images, it is possible to analyze the growth of individual cracks within the angle-ply laminates in more detail. In the following, crack interaction is analyzed using the experimental data gathered from the ${\left[0/+75_{2}/0/-75_{2}\right]}_{s}$, ${\left[0/+65_{2}/0/-65_{2}\right]}_{s}$, and ${\left[0/+55_{2}/0/-55_{2}\right]}_{s}$ laminates.

Crack spacings for several averaged crack densities were measured manually from the images of cracked angle-ply laminates acquired by the automatic procedure to investigate the different crack growth behavior in thin and thick layers. The averaged crack densities assume that all cracks have the same distance to their neighbors, hence, they have equidistant spacings. This research addresses whether cracks grow in areas of equidistant crack spacings or not, which is of high importance for any fatigue damage model to accurately perform stress or energy calculations when crack growth relations are established [36].

**Figure 14:**
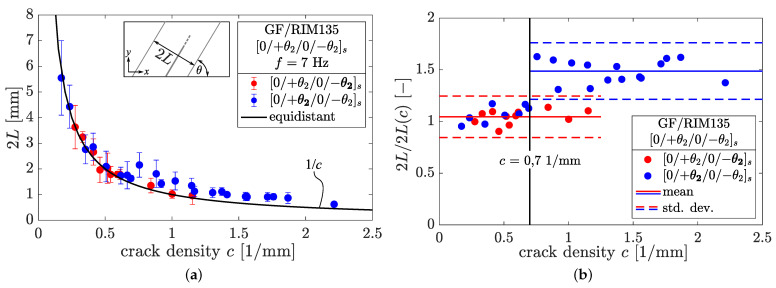
(**a**) Measured crack spacings of individual cracks compared to equidistant crack spacing as determined by the detection algorithm; (**b**) the ratio of real and equidistant crack spacing for several crack densities. Data were gathered from ${\left[0/+75_{2}/0/-75_{2}\right]}_{s}$, ${\left[0/+65_{2}/0/-65_{2}\right]}_{s}$, and ${\left[0/+55_{2}/0/-55_{2}\right]}_{s}$ laminates.

Therefore, the crack distance, 2 L, of two neighboring cracks next to a growing crack was measured for ten individual cracks from every image under consideration, with each image corresponding to a certain equidistant crack density. The results shown in Figure 14a were gathered from specimens with fiber angles of ±55°, ±65°, and ±75° for thick (−θ) and thin (+θ) layers, respectively. In total, six different specimens and 24 images taken over the whole range of loading cycles were analyzed. It was found that the measured crack spacings in the case of both thick and thin layers correspond very well with the equidistant crack spacing as long as $c<0.7{\mathrm{mm}}^{-1}$ holds true. Regarding crack densities of $c>0.7{\;\mathrm{mm}}^{-1}$, the measured crack spacings within the thin layers deviate from their corresponding equidistant crack spacings. Subsequently, the crack spacings of the thin layers in which crack growth takes place were larger for increasing crack densities compared to their corresponding equidistant values. In contrast, thick layers show good agreement between the measured and equidistant crack spacings for crack densities up to $c\approx 1.15{\;\mathrm{mm}}^{-1}$. Higher crack densities were been observed in thick layers in any of the experiments.

In Figure 14b, the ratio of the measured and equidistant crack spacing 2L/2L(c) is analyzed. It is confirmed that the ratio of measured to equidistant crack spacing is approximately constant for thick layers within the considered range. This means that the crack spacings measured by the automatic detection algorithm correspond well with the real crack spacing in which fatigue crack growth takes place. On the other hand, in the case of thin layers the ratio 2L/2L(c) changes significantly for crack densities $c>0.7\;{mm}^{-1}$, and increases to a mean value of approx. 1.5. This is in agreement with theoretical findings from Liu and Nairn [36], who used a scaling factor, *f*, in the range of 1 to 1.44 in order to appropriately account for crack formation at high crack densities.

Despite the particularly high scatter of the data, we found that the cracks tend to grow within bigger crack spacings. This clearly makes sense, as the effect of crack shielding is less pronounced within these regions and thus the normal and shear stresses within the uncracked regions between two cracks gain higher levels and promote fatigue crack growth.

In examining the transition point $c=0.7{\;mm}^{-1}$ more closely, the corresponding equidistant crack spacing is 2L=1/c=1.428 mm. When normalizing the crack spacing by means of the nominal +θ layer thickness of $t_{\theta }/2=t_{lam}/12\approx 0.18\;mm$, the dimensionless crack spacing $\rho =2L/t_{\theta }\approx 3.97$ can be determined. Krasnikovs and Varna [43] have previously pointed out that crack interaction has to be considered for ρ<4. Hence, fatigue crack growth in angle-ply laminates apparently shifts into regions without local crack interaction (lower local crack densities) when the existing cracks begin to interact. The transition point approximately coincides with the maximum crack density observed in static loading, while the maximum crack density observed during fatigue experiments is much higher compared to static loading. This is a remarkable difference between the two loading types, particularly because the maximum fatigue loads here were well below the maximum static loads.

## 5. Conclusions

The research presented here investigated the damage behavior of ${\left[0_{2}/90_{4}\right]}_{s}$ and three laminates consisting of ${\left[0/+\theta_{2}/0/-\theta_{2}\right]}_{s}$ lay-ups with θ={75°,65°,55°} in terms of matrix cracking for external uniaxial loading, yielding multiaxial stress states in the local plies. The aim of this work was to analyze several factors that influence fatigue crack initiation and growth. Additional static loading–unloading–reloading tests were conducted for reference.

Therefore, a TWLI methodology for the detection of matrix cracks was used in order to allow for the layer-wise determination of crack densities and the detailed analysis of individual crack growth. The validity of this method was checked by manual crack counting and microscopic analyses. This method is able to extract cracks that are situated next to bonding yarns with the same orientation as the cracks, and is therefore superior to manual counting.

The fatigue results were presented in terms of SN-curves for crack initiation, crack density evolution vs. load cycles, and cyclic stiffness loss. It can be stated that high load ratios lead to delayed crack initiation for each of the tested fiber orientations, while shear stresses promote crack initiation under fatigue loading. This is in agreement with previous findings by [14].

Based on our crack density evolution results, several effects which influence damage behavior under fatigue loading could be analyzed.

1. Increasing the maximum applied stress leads to faster and more pronounced evolution of cracks in fatigue loading due to higher stresses and resulting higher strain energy within the off-axis plies. The observed crack densities after $n=5\times 10^{5}$ loading cycles were between 0.25 to 1 cracks/mm for thick layers, while for thin layers the dependence on the load level was even more pronounced. In that case, the crack densities ranged from 0.15 to 2 cracks/mm.
2. High load ratios lead to less crack growth, even when the specimen is loaded with the same maximum stress, which is an effect of the smaller range of elastic strain energy stored in the material within a cycle. It is therefore recommended to adopt the range of the strain energy release rate, ΔG, as the driving force for cyclic crack growth.
3. Fiber orientations other than 0° and 90° lead to shear stresses which have a detrimental effect on crack growth in fatigue, leading to higher crack densities when experiments with nearly the same normal ply-stresses are compared. On the contrary, the detrimental effect has been observed in static loading, where lower fiber angles lead to less crack initiation and growth up to specimen failure. This might be due to increased fracture toughness from the higher mode mix.
4. Ply thickness affects the number of cracks forming in fatigue, and has little influence on fatigue crack initiation. This might be due to the bonding yarns, which can be seen as initial defects. Such defects trigger failure at very low loads and loading cycles.
5. In case of high crack densities (ρ≥4), individual cracks tend to grow within areas of locally larger crack spacings due to crack interaction. These crack spacings are on average 1.5 times the size of the corresponding equidistant crack spacing. This finding is in very good agreement with the theoretical estimations in [36], and provides experimental evidence for the physical meaning of scaling factors for crack spacings at high crack densities.

In the future, these results can serve as the basis for the development of an energy-based damage model for static and fatigue loading. Furthermore, the presented data can be used by other authors to establish or validate their own models.

## Figures and Tables

**Table 1:** Tensile material properties of glass fiber-reinforced composite material.

Parameter	$E_{11}$	$E_{22}$	$G_{12}$	$\nu_{12}$	$R_{11}$	$R_{22}$	$R_{12}$	$T_{g}$	$\alpha_{11}$ ^(1)^	$\alpha_{22}$ ^(1)^
Units	GPa	GPa	GPa	−	MPa	MPa	MPa	°C	10^−6^/K	10^−6^/K
μ	37.2	9.59	3.09	0.292	1059.6	33.6	48.9	83.7	8.25	38.90
σ	±0.3	±0.13	±0.12	±0.07	±19.9	±3.3	±0.7	±0.8	±0.45	±1.08
$n_{exp}$	5	4	5	5	5	4	5	6	4	4

^(1)^ at room temperature.

**Table 2:** Properties of GFRP laminates with multiaxial lay-ups.

Parameter	$t_{\mathrm{lam}}$	$\varphi_{f}$	$E_{\mathrm{xx}}$	$R_{\mathrm{xx}}$	$n_{\exp}$
Units	mm	%	GPa	MPa	−
${\left[0_{2}/90_{4}\right]}_{s}$	2.132±0.012	49.32±0.94	17.89±0.12	333.4±10.3	3
${\left[0/{+75}_{2}/0/{-75}_{2}\right]}_{s}$	2.171±0.028	47.89±0.58	17.10±0.04	318.4±0.6	3
${\left[0/{+65}_{2}/0/{-65}_{2}\right]}_{s}$	2.161±0.023	48.78±0.18	17.98±0.25	337.7±8.2	3
${\left[0/{+55}_{2}/0/{-55}_{2}\right]}_{s}$	2.147±0.017	48.73±0.25	16.88±0.29	351.6±7.5	3

**Table 3:** Testing schedule and stress states of off-axis layers for fatigue experiments.

Lay-Up	*R*	$\sigma_{\max}^{\mathrm{fat}}$ ^(1)^	$\sigma_{22}$	$|\tau_{12}|$	$\sigma_{22,\mathrm{res}}$	$|\tau_{12,\mathrm{res}}|$	$\lambda_{12}$
	−	MPa	MPa	MPa	MPa	MPa	−
${\left[0_{2}/90_{4}\right]}_{s}$	0.1	108	64.1	0.0	10.1	0.0	0.0
	0.5	92.4	57.2	0.0			0.0
	−0.1	75.6	47.9	0.0			0.0
		60 (102)	40.1 (61.1)	0.0 (0.0)			0.0 (0.0)
${\left[0/{+75}_{2}/0/{-75}_{2}\right]}_{s}$	0.1	123.3	69	11.7	10.7	0.5	0.170
	0.5	105.5	60.6	10.1			0.166
	−0.1	86.3	51.5	8.3			0.161
		68.5 (116.5)	43.0 (65.8)	6.7 (11.1)			0.156 (0.169)
${\left[0/{+65}_{2}/0/{-65}_{2}\right]}_{s}$	0.1	144	70.9	23.4	11.5	0.3	0.330
	0.5	123.2	62.3	20.1			0.323
	−0.1	100.8	53.1	16.5			0.311
		80 (136)	44.5 (67.6)	13.1 (22.1)			0.294 (0.327)
${\left[0/{+55}_{2}/0/{-55}_{2}\right]}_{s}$	0.1	162	60.7	36.9	12	0.7	0.608
	0.5	138.6	53.7	31.7			0.590
	−0.1	113.4	46.1	26			0.564
		90 ^(2)^	39.1	20.8			0.532

^(1)^ each load level was tested at all load ratios, unless otherwise stated. ^(2)^ not tested in case of *R* = 0.5. Values in () correspond to a load level of 0.85 × *σ_i_* tested at *R* = 0.5.

**Table 4:** Gabor filter parameters used for image filtering and image specifications.

Parameter	$\theta_{G}$	λ	$\rho_{G}$	β	$W_{I}$	$H_{I}$	γ
Units	°	px	px	−	px	px	px/mm
Value	θ− 90	12	10	0.11	2848	4288	128

**Figure A1:**
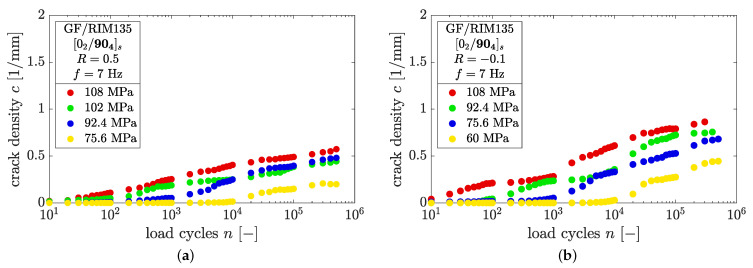
Results of the crack counting procedure for ${\left[0_{2}/90_{4}\right]}_{s}$-laminates for (**a**) R=0.5 and (**b**) R=−0.1.

**Figure A2:**
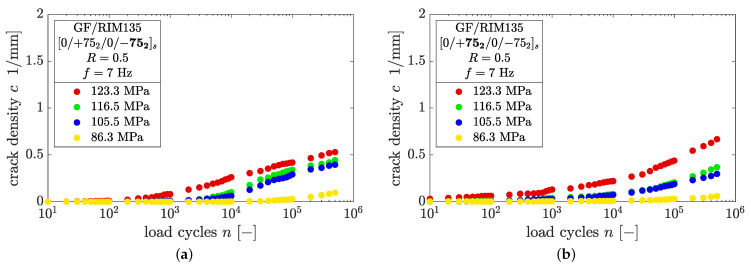
Results of the crack counting procedure for ${\left[0/{+75}_{2}/0/{-75}_{2}\right]}_{s}$-laminates for (**a**) thick and (**b**) thin layers at R=0.5.

**Figure A3:**
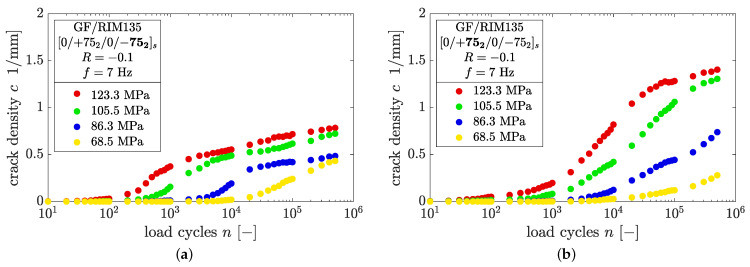
Results of the crack counting procedure for ${\left[0/{+75}_{2}/0/{-75}_{2}\right]}_{s}$-laminates for (**a**) thick and (**b**) thin layers at R=−0.1.

**Figure A4:**
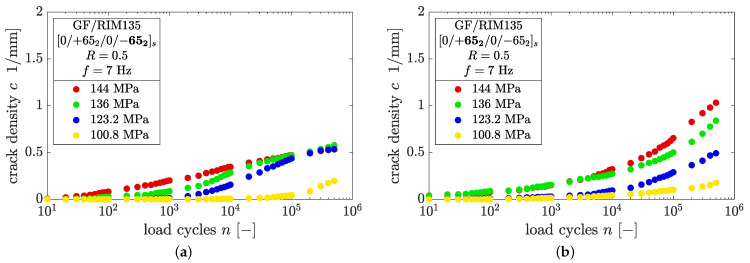
Results of the crack counting procedure for ${\left[0/{+65}_{2}/0/{-65}_{2}\right]}_{s}$-laminates for (**a**) thick and (**b**) thin layers at R=0.5.

**Figure A5:**
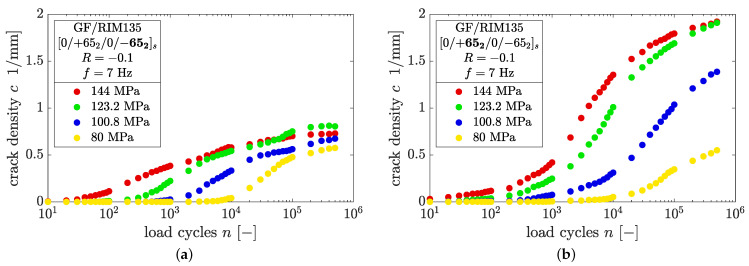
Results of the crack counting procedure for ${\left[0/{+65}_{2}/0/{-65}_{2}\right]}_{s}$-laminates for (**a**) thick and (**b**) thin layers at R=−0.1.

**Figure A6:**
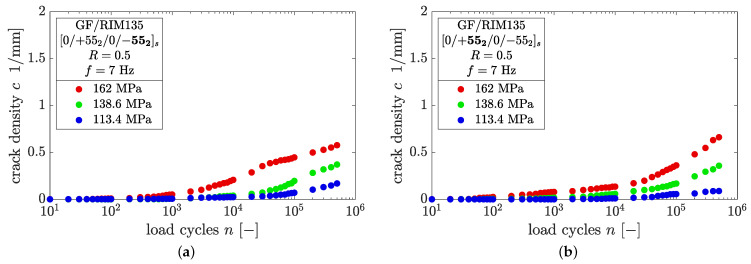
Results of the crack counting procedure for ${\left[0/{+55}_{2}/0/{-55}_{2}\right]}_{s}$-laminates for (**a**) thick and (**b**) thin layers at R=0.5.

**Figure A7:**
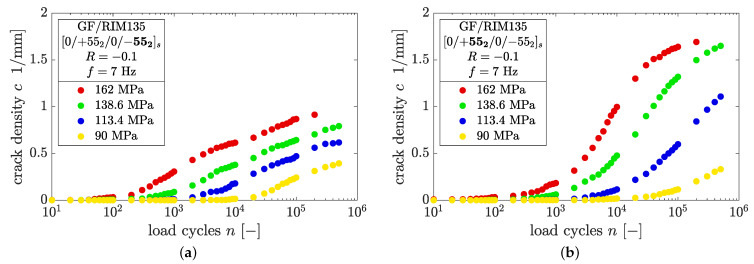
Results of the crack counting procedure for ${\left[0/{+55}_{2}/0/{-55}_{2}\right]}_{s}$-laminates for (**a**) thick and (**b**) thin layers at R=−0.1.

## Data Availability

Data available on request.

## Abbreviations

The following abbreviations are used in this manuscript:

FRP	Fiber-reinforced plastic
GF	Glass fiber
GFRP	Glass fiber-reinforced plastic
LUR	Loading–unloading–reloading
LVDT	Linear variable differential transformer
RTM	Resin transfer molding
TWLI	Transmitted white light imaging
UD	Unidirectional
VNRS	V-notched rail shear
